# Response to the letter to the editor: Evaluation of three inflammation-associated blood indices for predicting malignancy in thyroid nodules

**DOI:** 10.20945/2359-4292-2026-0070

**Published:** 2026-06-25

**Authors:** Merve Çatak, Bülent Koca, Zeynep Çetin, Özden Özdemir BAŞER

**Affiliations:** 1 Tokat Gaziosmanpasa University, Faculty of Medicine, Department of Endocrinology and Metabolism, Tokat, Türkiye; 2 Tokat Gaziosmanpasa University, Faculty of Medicine, Department of General Surgery, Tokat, Türkiye; 3 Amasya Sabuncuoğlu Şerefeddin Training and Research Hospital, Amasya Şerefettin Sabuncuoğlu, Faculty of Medicine, Department of Endocrinology and Metabolism, Amasya, Türkiye; 4 Dokuz Eylul University, Faculty of Medicine, Department of Endocrinology and Metabolism, Izmir, Türkiye

Dear Editor,

We would like to thank Öztürk for their interest in our study and for their thoughtful comments regarding the prognostic implications of inflammation-associated indices ^(1)^.

As correctly pointed out, regression-based models and receiver operating characteristic analyses are valuable tools for evaluating independent prognostic performance. However, we would like to clarify that the primary aim of our study was to investigate the diagnostic utility of systemic inflammatory indices in differentiating benign from malignant thyroid nodules, rather than their prognostic significance within malignant cases ^(2)^.

In our cohort, exploratory analyses performed within the malignant group did not reveal significant associations between the evaluated indices and tumor-related features. In accordance with standard statistical methodology, further multivariate analyses were not performed in the absence of significant findings in univariate analyses. Therefore, our findings should be interpreted as indicating that no significant prognostic association was identified in our dataset, rather than definitively excluding a possible prognostic role for these indices.

We also acknowledge that the relatively limited number of patients with advanced disease characteristics, particularly distant metastasis, may have reduced the statistical power to detect subtle prognostic associations. In this regard, previously published studies demonstrating the prognostic value of PIV in larger cohorts provide important contributions to the literature ^(3)^.

In conclusion, while our findings mainly support the potential diagnostic value of systemic inflammatory indices, further large-scale prospective studies focusing specifically on prognostic outcomes are needed to better clarify their potential clinical utility in thyroid malignancies.

## Data Availability

datasets related to this article will be avail-able upon request to the corresponding author.
